# Macroscopic irreversibility and microscopic paradox: A Constructal law analysis of atoms as open systems

**DOI:** 10.1038/srep35796

**Published:** 2016-10-20

**Authors:** Umberto Lucia

**Affiliations:** 1 Dipartimento Energia, Politecnico di Torino, Corso Duca degli Abruzzi 24, 10129 Torino, Italy

## Abstract

The relation between macroscopic irreversibility and microscopic reversibility is a present unsolved problem. Constructal law is introduced to develop analytically the Einstein’s, Schrödinger’s, and Gibbs’ considerations on the interaction between particles and thermal radiation (photons). The result leads to consider the atoms and molecules as open systems in continuous interaction with flows of photons from their surroundings. The consequent result is that, in any atomic transition, the energy related to the microscopic irreversibility is negligible, while when a great number of atoms (of the order of Avogadro’s number) is considered, this energy related to irreversibility becomes so large that its order of magnitude must be taken into account. Consequently, macroscopic irreversibility results related to microscopic irreversibility by flows of photons and amount of atoms involved in the processes.

In 1872, Boltzmann summarized his statistical mechanical results in his famous H-theorem. He introduced the irreversible evolution of any system towards a state of mechanical and thermal equilibrium. Loschmidt objected that this result is inconsistent, because any irreversible process cannot be obtained by using a time-symmetric dynamics^1^.

This controversy is no more than the problem of the link between the microscopic reversibility and the macroscopic irreversibility, named Loschmidt paradox. Despite the enormous advances of statistical mechanics in the description of equilibrium properties and transport processes in condensed matter, the problem of the non-contradictory microscopic foundation of both thermodynamics and kinetics remains unsolved^1^.

The analytical study of macroscopic irreversibility comes since 1789, when Benjamin Thompson (Count Rumford) highlighted that heat could be generated by friction^2^. In 1803, Lazare Carnot analyzed the conservation of mechanical energy for pulleys and inclined planes, pointing out that, in any movement, there always exists a loss of “moment of activity”^3^. But, the thermodynamic interpretation of this irreversibility was introduced first in 1824 by his son Sadi Carnot, who introduced the concept of the ideal engine, which is an ideal system which operates on a cycle in a completely reversible way, without any dissipation: unfortunately, efficiency of this ideal systems has an upper limit and isn’t unitary. Surprisingly, even in ideal condition without any dissipation, there is *something* that prevents the conversion of all the energy absorbed, from an ideal reservoir, into work^4^. This result was improved, in 1852, by Lord Kelvin, who pointed out that^5^,^6^:

1. The heat due to irreversibility is an irreversible process;
2. There is a universal tendency to the dissipation of mechanical energy into heat;
3. The heat wasted isn’t really wasted, but only a flux of heat from any open systems towards their environments.

In 1865, Rudolf Clausius introduced the *entropy* to analyse the dissipative processes^7^. The entropy obtained a statistical interpretation in 1872, just by Ludwig Boltzmann, as previously summarized. Boltzmann introduced the fundamental definition of statistical entropy, which, however, doesn’t consider any molecular correlation^8^,^9^. In 1889, Louis Georges Gouy, and, in 1905, Aurel Stodola, independently, proved that, in any process, the lost exergy (useful energy) is proportional to the entropy variation due to irreversibility^10^,^11^,^12^,^13^,^14^, providing a useful method to evaluate quantitatively the irreversibility. In 1902, it was Josiah Willard Gibbs^15^ to include molecular correlations among particles, pointing out that the total statistical entropy remains constant, and that something more must be included in the Boltzmann’s fundamental statistical results. In this context, Albert Einstein agreed with Gibbs’ viewpoint, and improved it by highlighting that the interaction of radiation with matter involves irreversible elementary processes in which total information results a conserved quantity^16^,^17^. In accordance with these considerations, Schrödinger introduced the hypothesis that the irreversibility is the result of interactions and fluxes between the systems and their surroundings: irreversibility has always a footprint which we evaluate as the growth of the entropy of the universe^18^.

All these results express the macroscopic irreversibility. But the experience of the macroscopic irreversibility is in contrast with a reversible model of the atomic structure. But, also at atomic level, irreversibility appears. Indeed, it was Planck to highlight how a single carbon particle would be enough to change perfectly arbitrary radiation into black radiation^19^. Moreover, it was pointed out that, when we consider the interactions between material particles and thermal radiation, the quantum mechanical analysis shows that particle path information isn’t preserved because the particle interactions with photons, in the thermal radiation fields, change the internal states of the particles themselves, with a related microscopical irreversibility^20^, in accordance with the irreversible measurements that John von Neumann showed to increase the entropy^21^. Recently, some experimental results have pointed out the quantum irreversibility^22^.

In this paper, we analyse the atoms and molecules as an open systems, and highlight a new approach to explain the macroscopic irreversibility in thermodynamics, based on the energy footprint due to the interaction of the atom with its environment^23^,^24^,^25^,^26^,^27^,^28^. For simplicity, but without lacking of generality, we analyse a Hydrogen-like atom^29^,^30^,^31^,^32^,^33^,^34^. The bases of our analysis is the Constructal law, the theory introduced to optimize the performance of thermo-fluid flow systems by generating geometry and flow structure, and to explain natural self-organization and self-optimization^35^,^36^,^37^,^38^,^39^,^40^,^41^,^42^,^43^,^44^. The developments of the Constructal law have offered a different look at corals, birds, atmospheric flow, and at machines in general. Here, we use this approach to study the foundation of atomic physics. We follow the Einstein’s, Schrödinger’s and Gibbs’ considerations on the interaction between particles and thermal radiation (photons), which leads to consider the atom as an open system in interaction with an external flows of photons. Our conclusions highlight how a single atom is no more than an open system and its interaction with the environment is irreversible. Last, in a single atomic transition, the energy related to the atomic irreversibility is negligible: microscopic irreversibility is very small in relation to the energy of interaction and the microscopic word appears reversible. On the contrary, when we consider an Avogadro’s number of particles, the energy related to irreversibility must be taken in account, and macroscopic irreversibility becomes evident. This paper represents also a first step towards the quantum Constructal law.

## Results

**Figure 1:**
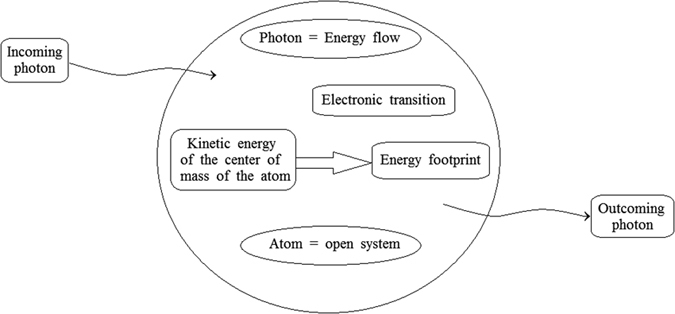
The atom as an open system.

The aim of this paper is to link the macroscopic irreversibility to the microscopic behaviour of atoms and molecules, by extending the Constructal law approach to quantum systems. These fundamental constituents of the matter are open systems, as represented in Fig. 1, because their electrons interact with external photons of the electromagnetic waves related to thermal radiation.

Constructal law is the thermodynamic theory based on the analysis of fluxes across the border of an open system. This theory is applied to finite size systems, and it is about their configurations. Here, we have highlighted that the atom is just an open system with energy level described by the principal quantum number *n*. The control volume used to develop the Constructal law analysis is a sphere: its center is the center of the atomic nucleus, and its radius is evaluated by the relation (11).

Following the Constructal law, the systems that prevail are the ones whose configuration and features evolve to facilitate flows through themselves in the least time. Here, following the Einstein’s approach, we have considered the electromagnetic wave as a flux of photons, which incomes into the atoms and molecules (our open systems). From a macroscopic point of view, the electromagnetic waves follow the Fermat’s least time principle, while from a microscopic point of view, at atomic level, Bohr described the absorption of a photon by an electron as a resonant process, at a maximum rate of absorption energy. The Bohr’s result means that the time of absorption of the incoming photons flux is minimum and, consequently, the open atom absorbs the energy flux, carried by the photons flux, in the least time.

At atomic level, the photons can be absorbed by the atomic or molecule electrons, and an electronic energy transition occurs between energy levels of two atomic stationary states. Then, the photons can be also emitted by the excited electrons when they jump down into the energy level of the original stationary state. During this phenomenon, the electrons seem to follow a reversible energetic path, because they come back to the original stationary state of low energy level. But, as a consequence of the interaction between the atomic or molecule electron and the photon, a footprint occurs in the atom or molecule. To analyse this phenomena, we consider the Constructal law.

Our result consists in pointing out that the interaction between a photon and an electron in an atom affects the energy level both of the electron and of the center of mass of the atom. When we consider a single atom or molecule, the energy perturbation of the center of mass is of the order of 10^−13^ J. A usual energy for the electron transition, between two atomic or molecule levels, is of the order of 10^−8^ J. Consequently, the order of magnitude of the microscopic energy footprint is of the order of 10^−5^–10^−4^ J, as evaluated for Hydrogen-like atoms in Table 1, where the energy footprint is evaluated by using the equation (24).

Consequently, when a single atom or molecule is considered, this atomic energy footprint, related to the atomic irreversibility, is negligible. But, when we consider a macroscopic system, we must consider the global effect of an Avogadro’s number of atoms. For example, if we consider the Earth mass (∼10^24 ^kg corresponding to ∼10^50^ atoms/molecules), the atomic energy footprint for electromagnetic interaction between atoms/molecules and photons results of the order of 10^45^–10^46^ J. This energy is lost by the Earth only for thermal disequilibrium, which causes a continuum electromagnetic interaction. But, this macroscopic irreversibility is no more than a consequence of the microscopic irreversibility.

Last, we have also highlighted how the irreversibility affects also the analytical description of the Schrödinger equation, as a consequence of the geometrical changes of the coordinates due to their relation with the momentum of the atoms or molecules, analytically proven by the equations (19–24).

## Discussion

When an electromagnetic wave interacts with the electrons of the atoms or of the molecules, its electromagnetic field changes the motion of the electrons, as a consequence of the energy absorbed or lost by the electron during the interactions. This atomic process can be well described by using the Bohr approximation^29^,^30^,^31^,^32^,^33^,^34^, as John Clarke Slater has highlighted^31^,^32^,^33^.

Bohr extended the Planck’s results describing the absorption of a photon by an electron as a resonant process, at a maximum rate of absorption energy. This means that the time of absorption of the incoming photons flux is minimum. This requirement is the same one of the Constructal law, so we can consider the photons flux as a flux of energy absorbed by the open system (in this case the atomic system) in the least time. This allows us to introduce the Constructal law in the analysis of atomic systems by a Bohr’s approach (semiclassical approach) to atoms, a first step to extend the Constructal law to quantum systems.

The result obtained is the consequence of considering the atom as an open system with continuous incoming and out-coming fluxes of photon. The consequence is that there exists a change in the kinetic energy of the center of mass of the atom or molecule. This is well known in atomic and molecular spectroscopy, but its amount is negligible in relation to the energy change in electronic transition and its time of occurrence (10^−13^ s) is greater than the time of electronic transition (10^−15^ s). On the contrary, considering a thermodynamic system, we consider a very large number of atoms and molecules, and the global effect of all these atomic and molecular energy changes become so relevant that the irreversibility appears. Consequently, macroscopic irreversibility is no more than the global superposition of the microscopic irreversibility, due to the large number of atoms and molecules in matter.

Last, we have proven that the microscopic irreversibility changes the Schrödinger equation of the atom after any photon-electron interaction. As a consequence, we can highlight that irreversibility is a constituent process of Nature, which means that the geometrical and analytical structures of the equations of the atomic processes, change as a consequence of the interactions with the surroundings.

Moreover, our results allow us to explain the Carnot’s results. Indeed, in the continuous interaction between electromagnetic waves and matter, any system loses energy for microscopic irreversibility and, consequently, any system cannot convert the whole energy absorbed into work.

## Methods

Constructal law is applied to finite size systems, and it is about features, configurations of such systems. The systems that prevail are the ones whose configuration and features evolve to facilitate flows through themselves in the least time. Consequently, we highlight as the atom is no more than an open system, and we must show the control volume considered for our analysis, in relation to the atomic and quantum properties. Then, we develop some considerations on the atomic configurations. Last, we highlight that the process of interaction between the electromagnetic waves (photons) and the atoms occurs in a way such that the process develops in the least time. Of course, some new viewpoint must be introduced in the Constructal law, because the process considered is a quantum process.

In 1911, after two years of analysis of the experimental results, Rutherford proved that the great part of the atomic mass is confined in a volume of 10^−15^ m radius, the nucleus. This result, together with the Earnshaw theorem on the unstable configuration of static charging points in a Coulomb electric field, led to the Bohr’s stationary states for the atoms, result useful to explain the experimental spectral lines of the electronic transitions in the atoms and molecules. Any atomic stationary state has a well defined energy level, identified by the principal quantum number^29^,^30^,^31^,^32^,^33^,^34^ *n*. An electronic transition between two energy levels can occur following the quantum selection rule^29^,^30^,^31^,^32^,^33^,^34^ Δ*n *= *n*_*f*_  −  *n*_*i*_ = ±1, where the subscript *f* means final state and the subscript *i* means initial state. Now, we consider an atom in interaction with external electromagnetic waves. For simplicity, but without lacking of generality, we analyse a Hydrogen-like atom^29^,^30^,^31^,^32^,^33^,^34^. We consider this atom as an open system because it interacts with its surroundings. So, in order to use the Constructal law, we must define the control volume considered. We introduce the Bohr’s model of atom, usually used in spectroscopy^30^,^32^,^34^: the atom has an atomic number *Z* and only one electron in the last orbital. This electron moves in its orbital with a momentum *p*_*e*_ = *m*_*e*_*v*_*e*_, where *m*_*e*_ is the mass of the electron and *v*_*e*_ its velocity inside the atom. For such electron, the Sommerfeld-Wilson rule states that:


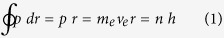


where *p r* is the angular momentum of the electron, being *r* the mean radius of a theoretical circular orbit of the electron in the orbital, *n *= 1, 2, 3, … is the principal quantum number, always integer, *h* is the Planck’s constant, and *ħ* is the Dirac constant (= 1.54571 × 10^−34^ J s). Moreover, the motion of the electron generates a centripetal force 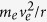 which is no more than the Coulomb force of the electric interaction between the nucleus and the electron:


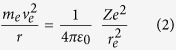


where *e* is the elementary charge (1.6 × 10^−19^ As), and *ε*_0_ is the electric permittivity (8.85419 × 10^−12^ F m^−1^). Moreover, the total energy of the atomic energy level results:


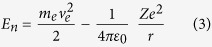


Now, considering the relations (1), (2), and (3), we can obtain:

1. The atomic radius:


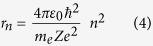


2. The energy of the atomic level:


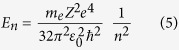


3. The velocity of the electron in the orbital:


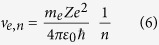


as a function of the principal quantum number *n*.

Now, considering an atom of principal quantum number *n*, we can define our control volume as the sphere of radius equal to *n *+ 1 with center in the center of the atomic nucleus.

We consider an atom in interaction with external electromagnetic wave. The electromagnetic waves carries:

1. An energy density evaluated as^30^:


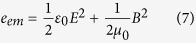


where *E* is the electric field, *μ*_0_ is the magnetic permeability (4π × 10^−7^ H m^−1^), and *B* is the magnetic field;

2. A momentum density evaluated as^30^:


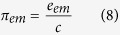


where *c* is the light velocity.

Since 1665, Newton introduced a corpuscular approach to light, but it was Einstein, in 1905, following the Planck’s results on the wave nature of particles, to develop the dual nature of light, introducing the concept of photon, as Lewis and Compton named the particle of light introduced by Einstein. The electromagnetic radiation is no more than a flux of photons, characterized by:

1. An energy *E*_*γ*_ evaluated as 30:


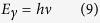


where *h* is the Planck’s constant (6.62607 × 10^−34^ J s), and *ν* is the frequency of the electromagnetic wave;

2. A momentum *p*_*γ*_ evaluated as:


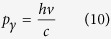


So, the interaction between the electromagnetic radiation and the Hydrogen-like atom can be studied as the interaction between the flux of photons with an open system (the atom of principal quantum number *n*), through the border of the control volume defined by the sphere of radius:


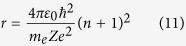


with center in the center of the atomic nucleus.

The time of the electronic transition is 10^−15^ s. Bohr extended the Planck’s results describing the absorption of a photon by an electron as a resonant process, at a maximum rate of absorption energy. The consequence of this result of Bohr is that the time of the electronic transition for electromagnetic interaction is the least time^30^,^31^,^32^.

As a consequence, the interaction between the electromagnetic wave and the atom is no more than the analysis of the interaction of the flux of photons across the border of the atomic control volume in the least time. But, this is just a typical case study of the Constructal law.

So, we can consider the interaction between the electromagnetic wave and the atom, just by using the Constructal law. An electron absorbs an incoming photon when its frequency *ν* is the resonant frequency (corresponding to the quantized energy), required by the transition between the initial *E*_*i*_ and final *E*_*f*_ energy levels^29^,^30^,^31^,^32^,^33^,^34^:


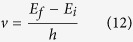


where *h* is the Planck’s constant. Emission of the this photon results in the reverse process. Now, we consider this process in the semiclassical approximation introduced by Bohr, as usually used in photochemistry and atomic/molecular spectroscopy.

Now, we begin considering the absorption process. At initial state, we consider the atom at rest, so its momentum **p**_*atm*_ is null. The momentum of the incoming photon is *hν* **u**_*c*_/*c,* where **u**_*c*_ is the versor of propagation of the electromagnetic wave, and *c* is the velocity of light in vacuum (2.9979 × 10^8^ m s^−1^). When an electron absorbs the incoming photon, the atomic momentum becomes:


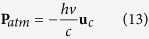


As a consequence of the absorption of the incoming photon, the electron undergoes an energy levels transition, from the stationary state of energy *E*_*i*_ to the stationary state *E*_*f*_, and the final energy of the atom can be evaluated by using the energy balance analysis and it results:


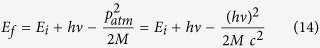


where *p*^2^_*atm*_/2*M* is the kinetic energy gained by the atom, and *M* is the mass of the atom. Consequently, we can obtain:


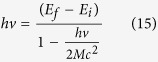


If we consider the reverse process, the emission of a photon, by using the same approach, we can obtain:


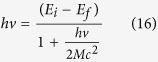


The two relations (4) and (5) present a correction term related to *hν*/2*Mc*^2^. This term can be evaluated considering that the energy of an electronic transition is of the order of 10^−13^ J, while the energy, *Mc*^2^, related to the mass of an atom *M* is of the order of 10^−8^ J. So, the correction term *hν*/2*Mc*^2^ is of the order of 10^−5^–10^-4^ J, negligible compared to 1 in the denominator, obtaining the well known relation used in spectroscopy^29^,^30^,^31^,^32^,^33^,^34^:


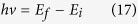


But, we must highlight that for a single atom we may not consider this correction because it is very small in relation to the transition energy, but we stress that this energy correction exists, and it is the energy footprint of the process.

In order to understand the fundamental origin of this energy footprint, we consider a Hydrogen-like atom and its Schrödinger’s equation, which is^29^,^30^,^31^,^32^,^33^,^34^:


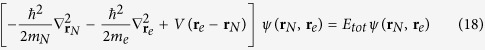


where *ħ* is the Dirac constant, *m*_*N*_ is the mass of the nucleus, *m*_*e*_ is the mass of the electron, **r**_*N*_ is the nucleus coordinate, **r**_*e*_ is the electron coordinate, *V*(***r***_*e*_ − ***r***_*N*_) is the electrostatic potential, *E*_*tot*_ is the total energy and *ψ*(***r***_*N*_, ***r***_*e*_) is the wave function. Now, introducing the relative coordinates ***r ***= ***r***_*N*_ − ***r***_*e*_, the coordinates of the center of mass ***R*** = (*m*_*N*_***r***_*N*_ + *m*_*e*_***r***_*e*_)/(*m*_*N*_ + *m*_*e*_), the total mass 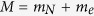, the reduced mass 
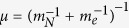
, the momentum of the center of mass 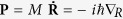, and momentum of the reduced mass particle 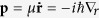, the equation (18) becomes^29^,^30^,^31^,^32^,^33^,^34^:


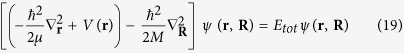


Now, we introduce the wave function *ψ*(***r***, ***R***) = *φ*(***r***)*ϑ*(***R***), in order to separate the equation (19) in the following two equations:


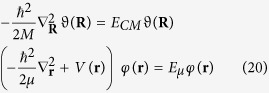


where *E*_*CM*_ = **P**^2^/2*M* is the energy of the free particle *center of mass*, and *E*_*μ*_ is the energy of the bound particle of *reduced mass*, such that *E*_*tot*_ = *E*_*CM*_ + *E*_*μ*_, and *V*(***r***) = −*Ze*^2^/*r*.

Now, we consider the absorption of the photon, as previously described, and the laws of conservation of momentum and energy due to the absorption of the photon, obtaining that:


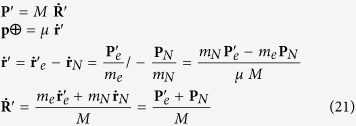


where **p**_*N*_ is the momentum of the nucleus and **p**_*e*_ is the momentum of the electron, 
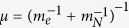
, and *M* = *m*_*e*_ + *m*_*N*_; now the Schrödinger’s equation becomes:


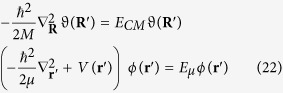


When the photon is emitted, following the same approach, we can obtain:


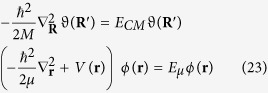


with an energy footprint


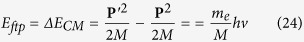


As a result, the correction of the energy of the center of mass corresponds to the energy footprint of the photon-electron interaction. In this way we have proven that a microscopic irreversibility exists, and it is a fundamental constituent of the geometrical structure of the Universe. The proof is based on the analysis of the fluxes by means of the Constructal law. This result can be also considered as a first step to introduce Constructal thermodynamics^45^ into the quantum and statistical physics.

## Additional Information

**How to cite this article**: Lucia, U. Macroscopic irreversibility and microscopic paradox: A Constructal law analysis of atoms as open systems. *Sci. Rep.* **6**, 35796; doi: 10.1038/srep35796 (2016).

## Figures and Tables

**Table 1:** The evaluation of the energy footprint ratio for the Hydrogen-like atoms.

Atom	*Z*	*m*_*e*_/*M*
**H**	1	5.44 × 10^−4^
**Li**	3	0.79 × 10^−4^
**Na**	11	0.24 × 10^−4^
**K**	19	0.14 × 10^−4^
**Rb**	37	0.06 × 10^−4^
**Cs**	55	0.04 × 10^−4^
**Fr**	87	0.04 × 10^−4^

The energy footprint ratio is the ratio between the atomic electron energy transition and the incoming or outcoming photon of the electromagnetic wave.
